# Respiratory Effects of Exposure to Traffic-Related Air Pollutants During Exercise

**DOI:** 10.3389/fpubh.2020.575137

**Published:** 2020-12-11

**Authors:** Giuseppe Morici, Fabio Cibella, Annalisa Cogo, Paolo Palange, Maria R. Bonsignore

**Affiliations:** ^1^Biomedicine, Neuroscience and Advanced Diagnostics Department, University of Palermo, Palermo, Italy; ^2^Institute for Biomedical Research and Innovation, National Research Council, Palermo, Italy; ^3^Biomedical Sport Studies Center, University of Ferrara, Ferrara, Italy; ^4^Department of Public Health and Infectious Diseases, Sapienza University of Rome, Rome, Italy; ^5^Department of Health Promotion Sciences Maternal and Infantile Care, Internal Medicine and Medical Specialties (PROMISE), University of Palermo, Palermo, Italy

**Keywords:** spirometry, biomarkers, exhaled nitric oxide, airway cell biology, performance, air quality

## Abstract

Traffic-related air pollution (TRAP) is increasing worldwide. Habitual physical activity is known to prevent cardiorespiratory diseases and mortality, but whether exposure to TRAP during exercise affects respiratory health is still uncertain. Exercise causes inflammatory changes in the airways, and its interaction with the effects of TRAP or ozone might be detrimental, for both athletes exercising outdoor and urban active commuters. In this Mini-Review, we summarize the literature on the effects of exposure to TRAP and/or ozone during exercise on lung function, respiratory symptoms, performance, and biomarkers. Ozone negatively affected pulmonary function after exercise, especially after combined exposure to ozone and diesel exhaust (DE). Spirometric changes after exercise during exposure to particulate matter and ultrafine particles suggest a decrease in lung function, especially in patients with chronic obstructive pulmonary disease. Ozone frequently caused respiratory symptoms during exercise. Women showed decreased exercise performance and higher symptom prevalence than men during TRAP exposure. However, performance was analyzed in few studies. To date, research has not identified reliable biomarkers of TRAP-related lung damage useful for monitoring athletes' health, except in scarce studies on airway cells obtained by induced sputum or bronchoalveolar lavage. In conclusion, despite partly counteracted by the positive effects of habitual exercise, the negative effects of TRAP exposure to pollutants during exercise are hard to assess: outdoor exercise is a complex model, for multiple and variable exposures to air pollutants and pollutant concentrations. Further studies are needed to identify pollutant and/or time thresholds for performing safe outdoor exercise in cities.

## Introduction

Air pollution is a major public health problem. In the United States, the 2019 State of the Air document highlighted the progressive recent worsening of air quality, and the strong and dangerous interaction between climate change and air pollution on respiratory health (1). According to the European Environment Agency (EEA), the air quality progressively improved over the years in Europe (2), but is still far from the World Health Organization standards. Moreover, during exercising in outdoor conditions, the exposure to pollutants may be magnified by the increased ventilation even at low pollutant concentrations (3).

Air pollution increases the risk for cardiorespiratory disease (4) and has been extensively studied in the general population and in subgroups at risk, i.e., children, elderly, or patients with cardiovascular or respiratory disease. However, the effects on the respiratory system of exposure to pollutants during exercise remain insufficiently characterized. High ventilation increases exposure and deposition rate of particles into the lungs during exercise (5), suggesting that athletes might be at particularly high risk. Olympic Games have been held in highly polluted areas, i.e., Beijing or Rio de Janeiro, requiring traffic limitation and shutdown of industrial plants to limit the effects of pollutants on athletes' health and performance (6, 7).

Habitual exercise is highly recommended to promote health (8). Accordingly, alternative transport strategies in cities have gained popularity. Active commuting, i.e., walking or biking to work or school, promotes a healthier lifestyle by increasing the amount of physical activity in the population while decreasing traffic volume (9). In some countries, like China, there is a strong tradition for outdoor exercise and few indoor facilities (10). It is recognized that increased exposure to traffic-related air pollution (TRAP), i.e., the air pollution originating from the emissions of motor vehicles and resulting from fossil fuel combustion, may exert negative effects on respiratory health (11).

We reviewed the literature on respiratory effects of TRAP and ozone exposure during exercise in normal subjects, general population including children and patients with chronic respiratory disease, and athletes. The details of the PubMed search strategy are reported in the Supplementary Material, together with the results of single studies organized according to the characteristics of the sample, exercise type, and indoor/field studies (Supplementary Tables 1–4). The following sections summarize the effects of air pollutants on (a) lung function tests, (b) subjective symptoms, (c) performance, and (d) biomarkers.

### Does Exercise in a Polluted Environment Affect Pulmonary Function Tests?

Exercise increases minute ventilation, magnifying the effects of the exposure to pollutants on the respiratory system (5). A meta-analysis on the effects of air pollution during exercise was inconclusive, since studies were few and involved a limited number of subjects, and the level of evidence was low or very low (12).

According to the majority of studies, air pollution worsened spirometric variables post-exercise, but the changes were quite small. Since bronchodilation occurs after moderate- to high-intensity exercise secondary to adrenergic receptor stimulation by circulating catecholamines (13), air pollutants appear to blunt the effects of exercise rather than causing a net decrease in lung function variables compared to baseline.

As for the effects of ozone exposure during exercise, decreased forced expiratory volume in one second (FEV_1_) and/or forced vital capacity (FVC) and/or peak expiratory flow (PEF) and/or forced expiratory flow between 25 and 75% of FVC (FEF_25−75_) were reported after prolonged exercise in hikers (14) and cyclists (15–17), whereas a short exercise and exposure duration may at least partly account for the nonsignificant results reported by a study in runners (18). Ozone exposure is highest during Summer, and an indoor study on the acute effects of ozone at 20 and 31°C in well-trained runners found no significant effect on respiratory function under either condition compared to no ozone exposure (19). Residential exposure to ozone and particulate matter (PM) may be more relevant in decreasing respiratory function in the long term compared to exposure to ozone or PM during exercise (20). Finally, children exercising outdoor in high-ozone areas showed an increased risk to develop asthma (21).

The level of exposure to ozone varied, since some studies were performed outdoors (14–16, 18, 20) while other studies used controlled exposure in the laboratory (17, 19). One study assessed the effects of combined acute exposure to diesel exhaust (DE) and ozone (DE+ ozone), documenting further worsening of spirometry compared to the effects of either pollutant alone; moreover, exposure to DE the day before was associated with worsened spirometry post-exercise during ozone exposure (22). Therefore, chronic exposure to pollutants may modulate the effects of subsequent acute exposures.

Data on the effects of TRAP exposure during exercise are heterogeneous, possibly because of the variable mixtures of pollutants. Pre-exercise exposure to DE for 60 min was associated with a lower increase in FEV_1_ post-exercise indoor compared to control conditions in cyclists (23), although DE had no effect on spirometry at rest. Field studies in subjects wearing sensors during exercise used black carbon (BC) measurements as indicative of TRAP exposure (24, 25). In short-term studies, increasing BC concentration in inspired air was associated with blunted exercise-induced bronchodilation and decreased PEF post-exercise in physically active normal subject (24). When the period of observation was extended to 1 week in three different seasons, a significant negative interaction was shown between physical activity and BC exposure on FEV_1_/FVC and FEF_25−75_, with a threshold at BC concentration > 1 μg/m^3^ (25).

Acute field studies in cyclists (26–28) or runners (29) reported worsened spirometry after exposure to a mixture of pollutants including PM of different sizes (PM_2.5_, PM_10_), NO_2_, BC, or ultrafine particulate matter (UFPM). High TRAP exposure before and/or during exercise was associated with decreased lung function (27). Some studies found an inverse relationship between pollutant concentrations and lung function (28, 29). However, other studies reported no significant changes in spirometric variables in studies in the laboratory (30, 31) or in field studies (32–34). Two studies assessed the level of pollutants by sensors on the bicycle (32, 33) leading to hypothesize that this methodology may lack sensitivity to reliably monitor respiratory exposure. The study by Kubesch et al. failed to show any significant interaction between intermittent moderate exercise and short-term exposure to pollutants (34). As for studies conducted in the laboratory, Bräuner used a complex protocol of 24-h continuous exposure to coarse PM; exercise tests were obtained after 15 min or 7.5 h of exposure. In this study, exercise intensity was moderate, and exercise tests were in the supine position (30).

One recent field study reported attenuated increase in FEV_1_ and FVC after a walk along a busy street compared to a walk in the park in middle-aged healthy controls; instead, FEV_1_ and FVC decreased post-exercise in COPD patients (35).

In summary, most studies indicated that acute exposure to air pollutants during exercise negatively affected respiratory function. The most recent studies have used complex designs to assess the interactions between the effects of exercise and air pollution. While exercise exerts positive effects on respiratory function variables, exposure to pollutants blunts the positive effects of exercise.

### Does Exercise in a Polluted Environment Cause Respiratory Symptoms?

Ozone exposure during cycling was associated with chest tightness (15, 17), shortness of breath, and wheezing (15). In cyclists, a high frequency of respiratory symptoms during and after commute in a highly polluted area was reported, especially by females and subjects with predisposition to respiratory diseases (36). Worse nasopharyngeal symptoms were reported during exposure to high pollutant levels during commute, and perception of pollution exposure was higher in females and the elderly (37). In studies testing the effects of DE exposure, increased fatigue not related to cycling intensity and respiratory symptoms were reported compared to the same exercise in filtered air (38, 39). Air pollution also worsened respiratory symptoms in COPD patients during a walk in a high-traffic area compared to a walk in the park (35).

Overall, while ozone caused respiratory symptoms in healthy subjects, possibly secondary to its irritant properties, respiratory symptoms did not usually occur in healthy people exercising under high TRAP conditions and were more common in females than in males. Exposure to TRAP worsened symptoms in patients with chronic respiratory disease.

### Does Exercise in a Polluted Environment Affect Performance?

A study analyzing the results of seven US marathons according to the levels of air pollutants reported worse marathon race times associated with increased PM10 levels, but the relationship was significant only in women (40). It was calculated that for every 10 μg/m^3^ increase in PM10 concentration, performance decreased by 1.4%. A lower performance was recorded in soccer players during exercise in a polluted vs. non-polluted area (41), in cyclists performing maximal exercise in the laboratory (42), and in children living in areas of high air pollution (43); in the latter study, level of SO_2_ exposure correlated inversely with exercise performance.

Conversely, other studies reported no effect of air pollution on performance. Acute exercise performance was similar in children from schools in areas of low and high exposure to pollutants (44). In runners during an 8-km test in the laboratory under different conditions of ozone and heat exposure, high temperature rather than ozone was associated with decreased performance (19). Performance during a 20-km ride in cyclists in the laboratory was unaffected by DE exposure (23).

In sedentary middle-aged healthy subjects training outdoor for 12 weeks in a rural or urban environment, fitness improved similarly in both groups, but the difference in UFPM exposure between the groups was small (mean UFPM concentration: 7,244 and 5,625/cm^3^ in urban and rural groups, respectively) (45).

The data suggest a strong need for additional studies especially in endurance athletes, in whom even small effects of air pollution may negatively affect competitive performances.

### Does Exercise in a Polluted Environment Affect Biomarkers?

Table 1 summarizes the results of several works reporting the assessment of different biomarkers of exposure, effect, or susceptibility after exercise under conditions of low and high exposure to pollutants. Most studies assessed changes in fractional exhaled NO (Fe_NO_), a marker of airway inflammation in asthma. Normally, Fe_NO_ increases after exercise (45) and is not an ideal indicator of pollutant-associated airway inflammation. Many studies reported unchanged Fe_NO_ after exercise during pollutant exposure (25, 34, 50) or similarly increased Fe_NO_ after exercise in filtered or polluted air (39); Strak et al. reported a slight increase (26), and Bos et al. showed a positive Fe_NO_ response to UFPM exposure during training (48). Only one study, assessing separately the alveolar and bronchial Fe_NO_, reported decreased alveolar Fe_NO_ after running in a highly polluted area (29). Finally, a recent long-term study using a complex methodology reported increased Fe_NO_ in urban runners undergoing frequent training sessions (46).

**Table 1:** Summary of the studies with measurements of biomarkers.

**Study, environmental condition (ref)**	**Exposure during exercise; measured pollutants**	**FeNO**	**EBC biomarkers**	**Alveolar-capillary membrane integrity**	**Inflammatory markers in blood and/or urine**	**Blood aromatic hydrocarbons**	**Plasma cytokines**	**Inflammatory cells in blood**	**BAL or sputum cells/markers**	**Induced sputum (IS) cell/markers**	**Cytokines in nasal lavage**
Chen 2018, outdoor exercise (46)	Long term; PM_2.5_, BC	↑ Especially if frequent exe	IL-1β=, IL-6=, IL-2=								
Giles 2018, cyclists (39)	Acute; DE (PM_2.5_ 300 μg/m^3^)	↑ After DE and control									
Laeremans 2018, cyclists (25)	Medium term in different seasons; BC	= After acute exe									
Cole 2018, cyclists (32)	Acute; PM_2.5_, PM_10_, PM_1_				CRP=, 8OHdG=		IL-6=				
Cavalcante 2016, runners (47)	Medium term; PM_2.5_, NO_2_, O_3_		pH trend to ↓								IL-8= IL-10=
Kubesch 2015, cyclists (34)	Acute; high-traffic environment	=			NOX=		IL-6=	WBC=, PMN=			
Bos 2013, running (48)	Long term; UFPM	↑						WBC ↑ PMN ↑			
Nwokoro et al., 2012, cyclists (49)	Long term; BC						IL-1beta=, IL-6=, IL-2=, IL-8= GM-CSF=, TNFα ↑ NS			↑ Area with BC in alveolar macrophages	
Jacobs 2010, cyclists (50)	Acute; O_3_	=		CC16= in serum			IL-6=	WBC = PMN ↑			
Blair 2010, runners (51)	Acute; high-traffic environment					↑ toluene, ethylbenzene and xylene					
Strak 2010, cyclists (26)	PM_10_	Weak ↑									
Bräuner 2009, cyclists (30)	24-h exposure; particle-rich air			DTPA=, CC16= in plasma and urine							
Chimenti 2009, runners (52)	Acute; PM_10_, NO_2_, O_3_								↑ PMN apoptosis	TNFα = IL-8 =	
Graff 2009, cyclists (31)	Acute; coarse PM								PMN ↑ 20 h post-exe	IL-6=, IL-8=, PGE2=, ↓ protein	
Ferdinands 2008, runners (53)	Acute; PM_2.5_, O_3_		pH=								
Rundell 2008, runners (29)	Acute; high pollution area	↓ alveolar eNO	↓NO_3_, ↑ MDA								
Bergamaschi 2001, cyclists (16)	Acute; high O_3_			↑ CC16 in serum							
Bergamaschi 1999, cyclists (54)	Acute; high-pollution area					↑Benzene and toluene in blood, ↑ toluene and xylenes in urine					
Kinney 1996, runners (55)	Long term; O_3_, PM_10_								↓ ROS and ↑LDH release by stimulated BAL cells in Summer		

Other studies assessed exhaled breath condensate (EBC) biomarkers. Two studies in runners reported unchanged EBC pH after exercise in clean or polluted air (47, 53). In runners, concentration of interleukin (IL)-1β, IL-6, or IL-2 in EBC was unchanged relative to exposure to air pollutants (46), while another study reported decreased nitrate and increased malondialdehyde (MDA) concentrations, suggesting increased oxidative stress in the airways during exercise in highly polluted areas (29). One study on nasal lavage found that IL-8 and IL-10 were unaffected by exposure to pollutants during exercise (47).

Analyses on plasma/serum or urine may provide biomarkers of pollutant-induced pulmonary damage. Integrity of the alveolar-capillary membrane, assessed as CC16 concentration in plasma or urine or 99m-Tc DTPA clearance, was unaffected after acute exercise under conditions of particle-rich air exposure (30). The effects of ozone in cyclists were variable, since post-exercise serum CC16 was increased in one study (16), and unchanged in another study (50). As inflammatory biomarkers are concerned, results were mostly not significant including measurements of cytokines (32, 34, 49, 50). Blood aromatic hydrocarbon concentrations were consistently increased in runners (51) and cyclists (54) exposed to TRAP during exercise. White blood cells (WBCs) and neutrophil (PMN) counts did not change in one study (34), whereas two studies reported increased PMN counts (48, 50) after exercise during exposure to pollutants compared to clean air.

Studies on cells recovered by bronchoalveolar lavage (BAL) or induced sputum yielded interesting results. A positive relationship between levels of PMN apoptosis in induced sputum and levels of PM_10_ and ozone exposure during exercise was found in runners (52). Another study found increased PMN in BAL collected 20 h after the end of exercise during exposure to coarse PM in cyclists (31). In urban bike commuters, the area of BC in alveolar macrophages was larger than in non-bike commuters when the sputum samples were examined at optical microscopy (49). A long-term study reported that during Summer, i.e., high ozone exposure, stimulated BAL cells released less radical oxygen species (ROS) and more lactic dehydrogenase (LDH) compared to samples collected in Winter (55).

More efforts should be done to improve our knowledge and understanding about the effects of air pollutant in outdoor athletes. There is some evidence in favor of increased oxidative stress and inflammation, but data are still insufficient to draw conclusions.

## Discussion

Detrimental effects of ozone and DE exposure on post-exercise spirometry were more often reported than no effects. Data on respiratory symptoms during exercise and performance similarly suggested a negative effect of air pollutant exposure, with a higher susceptibility in women compared to men. Fe_NO_, the most frequently measured biomarker of airway inflammation, was not affected by exposure to pollutants. Other inflammatory markers measured in blood showed little change. Studies assessing cell composition and characteristics in induced sputum or BAL produced interesting results, but due to the difficulty in performing such studies, their number was low and sample sizes were small.

The effects of exposure to pollutants during exercise are hard to assess. Outdoor exercise is a complex experimental model, due to exposure to multiple air pollutants, and variable pollutant concentrations according to site, prevalent winds, weather conditions, or time of the day (56), besides proximity to road and traffic intensity (3). Studies under real-life conditions are now simpler because of the availability of wearable sensors (57). In laboratory studies, a limited range of pollutant concentrations can be used during exposure, for ethical reasons. Finally, most studies used acute exercise, whereas the effects of air pollutants on the respiratory system may require prolonged exposures, and highly complex statistical modeling, to become detectable.

Studies in competitive athletes are scarce and regard highly selected subjects (58). Moreover, data during competitions are usually collected under conditions of low pollution, i.e., running races are held during the weekend, and with traffic restriction along the race track. No study has examined the effects of air pollution in athletes with regard to competition sites like stadium or tracks.

Habitual exercise in the general population exerts positive effects on health (8). Longitudinal studies in middle-aged or elderly subjects underlined the benefits of moderate habitual exercise on mortality, especially in subjects exposed to residential low-moderate levels of NO_2_ (59). In addition, NO_2_ exposure was positively associated with incident hospitalization for asthma and COPD, while physical activity reduced the risk to develop asthma or COPD (60).

**Figure 1:**
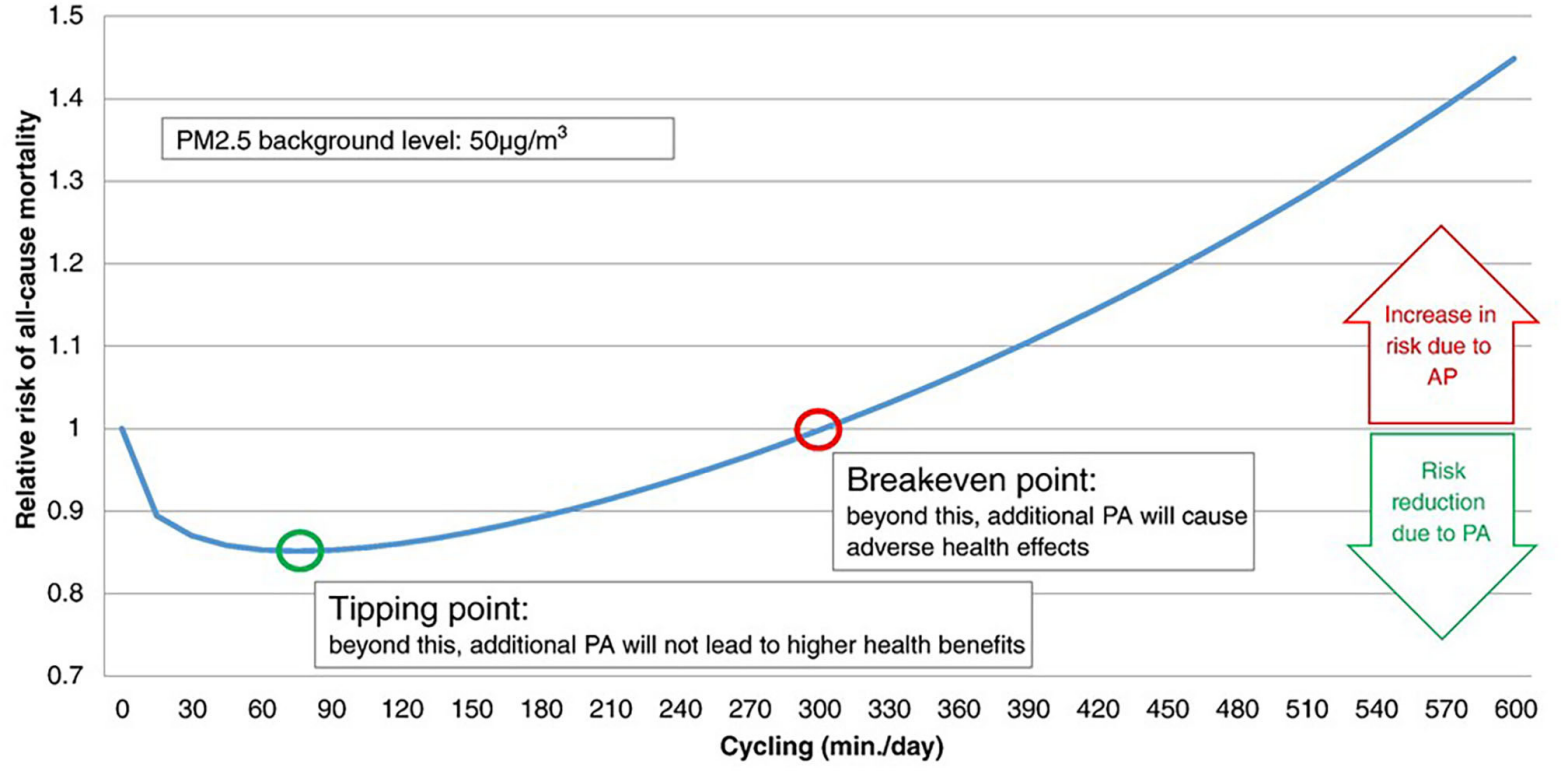
Relative risk (RR) for all-cause mortality (ACM) when physical activity (cycling) occurs during exposure to air pollution (at 50 μg/m^3^ PM_2.5_). The tipping point and break-even point indicate the lowest risk, and the lack on any protective effect of exercise, respectively. According to this model, the tipping point is at cycling for <90 min/day, while the break-even point indicates that any benefit of cycling is lost for >5 h/day cycling. Reproduced from Tainio et al. (61), used by permission under Creative Commons license.

The pros and cons of active transport to commute to study or work, i.e., cycling and walking in healthy people (9), are being extensively studied. In a study comparing exposure to TRAPs during different commuting modes, the relatively low exposure during cycling was outweighed by the increased ventilation during exercise (3). An interesting model calculated the all-cause mortality risk for air pollution exposure during active transport, with special attention to two points (Figure 1). The “tipping point” represents the amount of cycling per day associated with the highest risk reduction; the “break-even point” represents the amount of cycling per day beyond which increasing the amount of cycling is associated with increased pollution-related risk (61). While physical activity should be of short duration in heavily polluted cities, the model suggests no major harm associated with active transport, unless heavy pollution occurs, or exercise lasts several hours per day (61). Therefore, regular active commuting may cause some pulmonary damage, and the problem may be relevant for professional “riders.”

Analysis of pro-inflammatory effects of air pollutants during exercise is complicated by the effects of exercise *per se* on airway inflammation, especially in endurance athletes (62, 63). High concentrations of hydrocarbons were found in the blood of urban runners (51), while results on biomarkers in EBC have been disappointing to date. Studies on induced sputum or BAL found evidence of increased deposition of BC in macrophages of commuter cyclists than in non-cyclists (49) and increased oxidative stress or neutrophil apoptosis related to exposure to ozone or PM_10_ (52).

As for sex-related susceptibility to the effects of air pollutants (64), women reported more pollutant-induced respiratory symptoms than men (36, 37). These findings are in line with decreased FEV_1_ and FVC in women, but not in men, living in high-traffic areas (65), and with higher prevalence of respiratory symptoms in women exposed to moderate traffic (66). Women reported more allergic symptoms than men when chronically exposed to air pollutants (67). Symptoms may also be secondary to sex-related differences in work of breathing during exercise (68).

Common-sense strategies can limit the impact of air pollution, especially during exercise (69). A meta-analysis on the interaction between air pollution and physical activity, based on seven studies in over 50,000 subjects, reported an inverse relationship between PM_2.5_ levels and physical activity (70), reflecting a preventive behavior by the exposed population. A healthy diet and vitamin C and D supplementation could help limit air pollution-related respiratory damage (71). Finally, the possibility that healthy well-trained athletes may develop protective defense mechanisms has been not explored, although some studies highlighted differences in response to air pollutant exposure during exercise linked to specific genetic background regarding antioxidant and anti-inflammatory responses (16, 54).

In conclusion, most studies agree on the benefits of exercise in healthy subjects even during increased exposure to air pollution. Since a large fraction of the world population lives in cities, protection of respiratory health is mandatory, and a safe environment for physical activity is highly desirable. For these reasons, further research is needed for overcoming the present variability in results, for clearly defining the effect of urban pollution on regular active commuting, and for developing laboratory settings able to focus on cellular models and thus to investigate more clear cause–effect relationships. Finally, the development of “personal” monitors able to reliably measure exposure at the individual level will make possible longitudinal “real life” studies.

## Author Contributions

GM, FC, and MB conceived the manuscript, did the searches, and wrote the draft of the manuscript. AC and PP critically revised the manuscript and contributed to the discussion. All authors contributed to the article and approved the submitted version.

## Conflict of Interest

The authors declare that the research was conducted in the absence of any commercial or financial relationships that could be construed as a potential conflict of interest.

## Supplementary Material

Supplementary Text PubMed search strategy.

Supplementary Figure 1 Summary of PubMed search strategy.

Supplementary Table 1 Meta-analyses and field studies in the general population.

Supplementary Table 2 Studies with controlled exposure to pollutants during exercise- in-laboratory studies.

Supplementary Table 3 Field studies in cyclists.

Supplementary Table 4 Field studies in runners, soccer players and hikers.
