# Supplementary material for: Respiratory Effects of Exposure to Traffic-Related Air Pollutants During Exercise
Source: Front Public Health. 2020 Dec 11;8:575137. doi: 10.3389/fpubh.2020.575137 (PMC7793908; doi:10.3389/fpubh.2020.575137)
Supplement: Supplementary Text — PubMed search strategy. [file Data_Sheet_1.docx]

**Methods**

Studies in English published since 1987, reporting data collected in human subjects during exercise outdoor or during controlled exposure to pollutants in the laboratory, were reviewed. Report of exercise data and exposure data was required for inclusion. PubMed was used as the database for search (November 2018, updated on Oct 25, 2019). Relevant additional studies, cited by selected papers but not resulting from the search, were also included. Two searches were performed using the following terms:

1. Sport, air pollutants, and respiratory ("sports"[MeSH Terms] OR "sports"[All Fields] OR "sport"[All Fields]) AND "air pollutants"[All Fields] AND "respiratory"[All Fields]);
2. Sport, air pollutants and biomarkers ("sports"[MeSH Terms] OR "sports"[All Fields] OR "sport"[All Fields]) AND "air pollutants"[All Fields] AND "biomarkers"[All Fields]).

Studies on moderate to heavy exercise during exposure to air pollutants vs filtered air, or in areas with high- vs low-pollutant concentrations were selected, or studies on intermittent exercise with overall exercise duration of at least 30 min. Such duration was chosen because shorter exercise bouts were used in earlier studies to increase exposure to pollutants, without specific assessment of respiratory variables during/after exercise (1, 2).

Review articles, papers not pertinent with regard to questions, or without respiratory or exposure data were excluded. Papers reporting the effects of swimming or Winter sports were also excluded, since they refer to athletes performing under peculiar environmental conditions, i.e. exposure to chlorine-derived pollutants or pollutants present in ice arenas. These sports were analyzed in a recent review (3).

Each paper was reviewed by GM and MRB and the following data were extracted: type of study, subjects involved (sample size, sex and age), protocol, exposure to pollutants, outcome measures and results. Figure 1 illustrates the selection process, yielding 45 articles included for qualitative analysis. The Supplementary Tables report details on each included article, according to the characteristics of the sample, exercise type and indoor/field studies, as well as the references cited in each Table:

Table S1: meta-analyses and population field studies;

Table S2: Studies with controlled exposure to pollutants during exercise in the laboratory;

Table S3: Field studies in cyclists;

Table S4: Field studies in runners, soccer players and hikers).

References

1. Holgate ST, Sandström T, Frew AJ, *et al*. Health effects of acute exposure to air pollution. Part I: Healthy and asthmatic subjects exposed to diesel exhaust. Res Rep Health Eff Inst. 2003 Dec;(112):1-30; discussion 51-67.
2. Holgate ST, Devlin RB, Wilson SJ, Frew AJ. Health effects of acute exposure to air pollution. Part II: Healthy subjects exposed to concentrated ambient particles. Res Rep Health Eff Inst. 2003 Dec;(112):31-50; discussion 51-67
3. Rundell KW, Smoliga JM, Bougault V. Exercise-induced bronchoconstriction and the air we breathe. Immunol Allergy Clin North Am. 2018; 38(2):183-204.
