# Supplementary figures and images for: Respiratory Effects of Exposure to Traffic-Related Air Pollutants During Exercise
Source: Front Public Health. 2020 Dec 11;8:575137. doi: 10.3389/fpubh.2020.575137 (PMC7793908; doi:10.3389/fpubh.2020.575137)

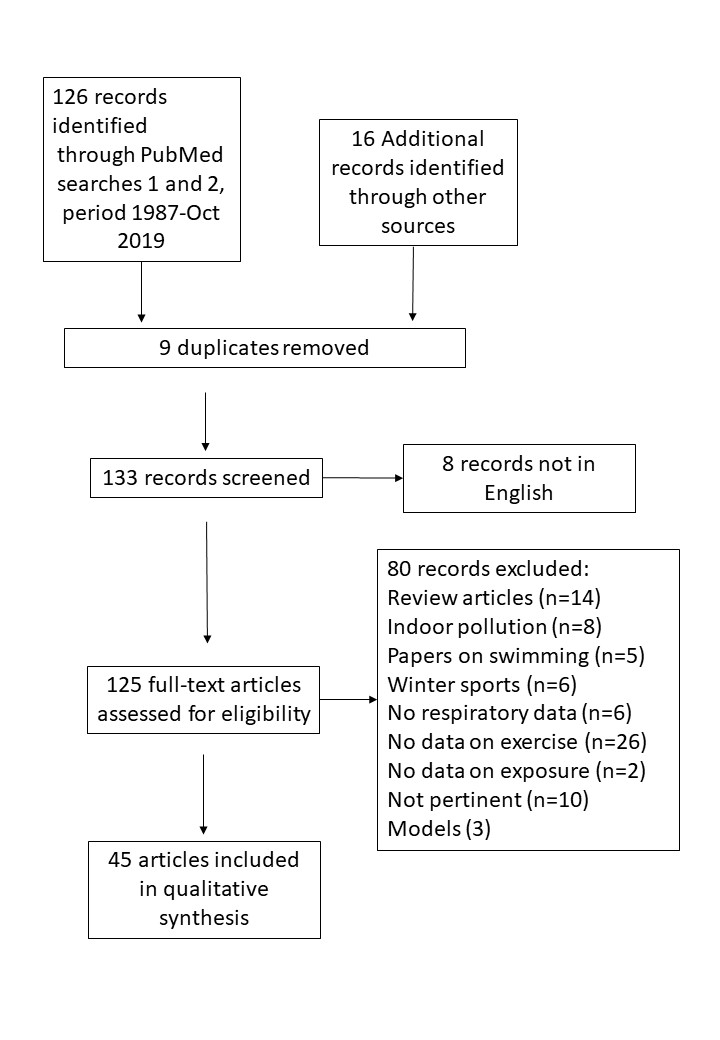

Supplement: Supplementary Figure 1 — Summary of PubMed search strategy. [file Image_1.JPEG]
