# Supplementary material for: Respiratory Effects of Exposure to Traffic-Related Air Pollutants During Exercise
Source: Front Public Health. 2020 Dec 11;8:575137. doi: 10.3389/fpubh.2020.575137 (PMC7793908; doi:10.3389/fpubh.2020.575137)
Supplement: Supplementary Table 1 — Meta-analyses and field studies in the general population. [file Table_1.DOCX]

| **Table S1. Meta-analysis and general population field studies** | | | | | |
| --- | --- | --- | --- | --- | --- |
| **Authors** | **Type of study** | **Subjects** | **Exposure** | **Outcomes** | **Key findings** |
| An et al, 2018 (1) | Meta-analysis | Over 50,000 subjects for effects of air pollution on physical activity in USA and UK. Seven studies met the inclusion criteria, 2 studies were on cardiopulmonary patients. | PM2.5, PM10, O3, NOx | Meta-analysis of the effects of PM2.5 on physical inactivity | In general, air pollution level associated negatively with physical activity and positively with leisure-time physical inactivity, especially in patients with cardiopulmonary disease. One unit (μg/m3) increase in ambient PM2.5 concentration was associated with an increase in the odds of physical inactivity by 1.1% |
| Qin et al, 2018  (2) | Meta-analysis | Articles that evaluated the combination effect of air pollution exposure and exercise on health. | O3, diesel exhaust (DE), traffic-related air pollution, particulate matter (PM) | Meta-analysis of the effects of exercise in a polluted air environment on lung function | Only peak expiratory flow (PEF) was found to be significantly decreased after exercise in polluted environment. |
| Chen et al, 2018 (3) | Observational repeated-measure study | 20 healthy young subjects undergoing multiple visits during Winter 2014 (heating season) in Bejing. Information on health and exercise habits (outdoors vs indoor, number of sessions/week) collected. | Monitoring of air quality, mean exposure to:  - PM2.5 : 65.2 ± 69.6 μg/m^3^  - Black carbon (BC): 5.3 ± 4.2 μg/m^3^  Number of ultrafine particles (UFP): 16.2 ± 7.3•10^3^/cm3; number of accumulation mode particles (AMP): 4.6 ± 3.8 | FeNO, cardiovascular variables, interleukin(IL)-1β, IL-2 and IL-6 in exhaled breath condensate (EBC) | Increased FeNO with increased PM2.5 and AMP exposure, especially in subjects with high exercise frequency. Unchanged cytokines in EBC |
| Sinharay et al, 2018 (4) | Observational cross-over study | 40 COPD patients, age ≥ 60 years, FEV_1_/ FVC < 0.7 and FEV_1_ ≤80% of predicted. Patients with ischemic heart disease (n=39) and healthy controls (n=40) were also studied.  . | Mean exposure level in Oxford St compared to Hyde Park: BC: 5-fold increase; PM10: increased by about 30%; PM2.5: 3-fold increase; ultrafine particles: 4-fold increase; and nitrogen dioxide (NO2): 4-fold increase | Differences in spirometry between 2-h walk in the study sites (commercial street, and urban park). | In all participants, walking in Hyde Park led to an increase in lung function (FEV1 and FVC) up to 26 h after the walk. Such responses were attenuated after walking in Oxford St. In participants with COPD, exposure to NO2, ultrafine particles and PM2.5 while walking led to respiratory symptoms and reduction in FEV1 and FVC. |
| Fisher et al, 2016 (5) | Longitudinal cohort study | 57.053 Danish subjects (Danish Diet, Cancer, and Health cohort) aged 50–64 years. Subjects reported physical activity at recruitment (1993-1997). Follow-up until 2013. | Levels of NO2 were estimated at subjects’ residence at the time of recruitment. Exposure to NO2 defined as high: >21.0 mg/m^3^, medium: 14.3–21.0 mg/m^3^, or low: <14.3 mg/m^3^ | Incident hospitalizations for asthma or COPD | 1,151 subjects were hospitalized for asthma and 3,225 for COPD during 16 years. NO2 exposure was positively associated with incident asthma and COPD hospitalizations, and inversely associated with participation in sports. No interaction between physical activity and NO2 on incident asthma or COPD hospitalizations. Increased exposure to air pollution during exercise did not outweigh beneficial effects of physical activity on the risk of asthma and COPD. |
| Andersen et al, 2015 (6) | Longitudinal cohort study | in 52.061 Danish subjects (Danish Diet, Cancer, and Health cohort) aged 50–65 years. Data on physical activity (participation in sports, cycling, gardening, and walking) were collected at recruitment (1993–1997). Follow-up until 2010. | Levels of NO2 were estimated at subjects’ residence at the time of recruitment.  NO2 exposure classified as: 1) high versus moderate/low exposure: ≥ 19.0 vs. < 19.0 μg/m^3^; 2) exposure levels: very high >23.9 mg/m^3^, moderate 15.1–23.9 mg/m^3^, low <15.1 mg/m^3^ | Mortality by Cox regression, NO2 used as an interaction term. | Total mortality (n=5,534): cancer (n=2,864), cardiovascular disease (n=1,285), respiratory disease (n=354), and diabetes (n=122). Decreased respiratory mortality was associated with cycling and gardening, especially among participants with moderate/low NO2 exposure compared to participants exposed  to high NO2. |
| Ramirez et al, 2012 (7) | Cross-sectional cohort study | 1,045 children aged 7-12 years attending 4 schools in Bogotà, undergoing 20 m shuttle-run test and spirometry (n=434) | One school in slightly polluted area (PM10 mean concentration 55.3 μg/m^3^); 3 schools in highly polluted areas (PM10 mean concentrations: 90.5, 87.8 and 90.4 μg/m^3^) | Calculated V’O2max in the entire sample. | No difference in acute performance between children attending school in a slightly polluted compared to highly polluted areas |
| Zebrowska & Mankowski, 2010 (8) | Cross-sectional cohort study | 103 healthy adolescents  (48 girls and 55 boys) aged 14-16 years from Upper Silesian Industrial region, undergoing cycle ergometer and running tests. | Average annual pollutant (SO2, NO2, PM10, CO) concentrations measured in the years 1994-2008. | Standard spirometry and maximal  voluntary ventilation (MVV), performance | Exposure to air pollution was associated with reduced respiratory function and performance in exercise tests. The difference between predicted and measured results of exercise tests correlated with increased SO2 concentration. No correlation between long-term exposure to air pollutants and estimated VO2max. |
| McConnell et al, 2002 (9) | Longitudinal cohort study | 3,535 children without asthma at baseline, living in cities in Southern California, with high- (H) or low- (L) pollution levels. Children were recruited in schools and participation to team sports was recorded | Mean exposure to pollutants over 4 years:  O3: L 40.0 ppb, H 59.6 ppb; PM10: L 21.6 mg/m^3^, H 43.3 mg/m^3^; PM2.5: L 7.6 mg/m^3^, H 21.4 mg/m^3^; NO2: L 10.8 ppb, H 29.2 ppb. | Incident asthma according to pollution level and sport activities. | Children playing 3 or more sports in areas of high O3 showed a 3-fold risk of developing asthma. Time spent outdoor also increased the risk of asthma in area of O3. No effect was shown in areas of low O3. Other pollutants did not affect asthma risk. |

References

1. An R, Zhang S, Ji M, Guan C. Impact of ambient air pollution on physical activity among adults: a systematic review and meta-analysis. Perspect Public Health. 2018;138(2):111-121. doi: 10.1177/1757913917726567.
2. Qin F, Yang Y, Wang ST, Dong YN, Xu MX, Wang ZW, Zhao JX. Exercise and air pollutants exposure: A systematic review and meta-analysis. Life Sci. 2019 Feb 1;218:153-164. doi: 10.1016/j.lfs.2018.12.036. Epub 2018 Dec 21. PubMed PMID: 30582950.
3. Chen X, Chen W, Wang Y, Han Y, Zhu T. Responses of healthy young males to fine-particle exposure are modified by exercise habits: a panel study. Environ Health. 2018 Dec 13;17(1):88. doi: 10.1186/s12940-018-0437-3. PubMed PMID: 30545423; PubMed Central PMCID: PMC6293663.
4. Sinharay R, Gong J, Barratt B, Ohman-Strickland P, Ernst S, Kelly FJ, Zhang JJ, Collins P, Cullinan P, Chung KF. Respiratory and cardiovascular responses to walking down a traffic-polluted road compared with walking in a traffic-free area in participants aged 60 years and older with chronic lung or heart disease and age-matched healthy controls: a randomised, crossover study. Lancet. 2018;391(10118):339-349. doi: 10.1016/S0140-6736(17)32643-0.
5. Fisher JE, Loft S, Ulrik CS, Raaschou-Nielsen O, Hertel O, Tjønneland A, Overvad K, Nieuwenhuijsen MJ, Andersen ZJ. Physical activity, air pollution, and the risk of asthma and chronic obstructive pulmonary disease. Am J Respir Crit Care Med. 2016;194(7):855-865.
6. Andersen ZJ, de Nazelle A, Mendez MA, Garcia-Aymerich J, Hertel O, Tjønneland A, Overvad K, Raaschou-Nielsen O, Nieuwenhuijsen MJ. A study of the combined effects of physical activity and air pollution on mortality in elderly urban residents: the Danish Diet, Cancer, and Health Cohort. Environ Health Perspect. 2015;123(6):557-563. doi: 10.1289/ehp.1408698.
7. Ramírez A, Sarmiento OL, Duperly J, Wai Wong T, Rojas N, Arango CM, Maldonado A, Aristizabal G, Pérez L, Lobelo F. Should they play outside? Cardiorespiratory fitness and air pollution among schoolchildren in Bogotá. Rev Salud Publica (Bogota). 2012 Aug;14(4):570-83. PubMed PMID: 23912511.
8. Zebrowska A, Mankowski R. Effects of long-term exposure to air pollution on respiratory function and physical efficiency of pre-adolescent children. Eur J Med Res. 2010;15 Suppl 2:224-8.
9. McConnell R, Berhane K, Gilliland F, London SJ, Islam T, Gauderman WJ, Avol E, Margolis HG, Peters JM. Asthma in exercising children exposed to ozone: a cohort study. Lancet. 2002;359(9304):386-391.
