# Supplementary material for: Respiratory Effects of Exposure to Traffic-Related Air Pollutants During Exercise
Source: Front Public Health. 2020 Dec 11;8:575137. doi: 10.3389/fpubh.2020.575137 (PMC7793908; doi:10.3389/fpubh.2020.575137)
Supplement: Supplementary Table 2 — Studies with controlled exposure to pollutants during exercise- in-laboratory studies. [file Table_2.DOCX]

| **Table 2. Exercise tests in the laboratory** | | | | | |
| --- | --- | --- | --- | --- | --- |
| **Authors** | **Type of study** | **Subjects** | **Exposure during exercise** | **Outcomes** | **Key findings** |
| Giles et al, 2018 (1) | Randomized repeated-measure study | 18 males (mean age 24.5 years) during cycling for 30-min at low- and high-intensity (30% and 60% of power at VO2peak, respectively) | Filtered air (FA, control) and diesel exhaust (DE) exposure containing 300 µg/m^3^ of PM2.5 | Respiratory symptoms, FeNO | More respiratory symptoms after DE than FA exposure. FeNO increased similarly after exercise irrespective of DE or FA exposure |
| Giles et al, 2014 (2) | Randomized repeated-measure study | 18 males (mean age 24.5 years) studied at rest and during cycling for 30 min at low- and high-intensity (30% and 60% of power at VO2peak, respectively). | Filtered air (FA, control) and diesel exhaust (DE) exposure containing 300 µg/m^3^ of PM2.5 | Respiratory and metabolic responses, and perceived exertion for lung and legs (Borg scale) during exercise | Low-intensity exercise: Respiratory and metabolic responses larger in DE compared to FA. Mean V’E (44.5 vs 40.5 L/min); V’O2 (27.9 vs 24.9 mL/kg/min); V’CO2 (25.9 vs 23.5 mL/kg/min)  High-intensity exercise: no significant difference between DE and FA  Increased perceived exertion for lungs and legs during DE exposure, without interaction exposure x intensity |
| Madden et al, 2014 (3) | Randomized cross-over study | 15 healthy, nonsmoking volunteers (4 F, 11 M, mean age 27 years) undergoing a 2-day protocol, with intermittent exercise (15 min rest - 15 min recumbent cycling for 2 h) during exposure to pollutants | Day 1: exposure to FA, or diesel exhaust (DE) 300 µg/m^3^, or O3 0.300 ppm, or DE 300 µg/m^3^ +O3 0.300 ppm  Day 2: exposure to O3 0.300 ppm  Day 3: follow-up  Exercise workload during day 1 and day 2 adjusted to reach ventilation of 25 L • min^-1^ • m^2^BSA | FEV1 and FVC measured before exposure + exercise, and for 4 h after exposure each day | On day 1, FA or DE alone did not affect FEV1 and FVC, but O3 decreased FEV1, and DE+O3 induced a greater FEV1 decrement.  On day 2, compared to previous exposure to FA, previous DE exposure worsened the FEV1 and FVC response to O3 exposure. |
| Giles LV et al, 2012 (4) | Randomized cross-over study | Eight endurance-trained men (mean age 29 years) undergoing two 20-km cycling time trials | Pre-exercise 60-min exposure to filtered air (FA) or diesel exhaust (DE, 300 µg/m^3^ of PM2.5) | Spirometry and measurement of ventilatory and metabolic variables during exercise, performance | Spirometry unaffected by DE exposure at rest. Lower increase in FEV1 after DE compared to filtered air, while respiratory variables or performance were unaffected |
| Cutrufello et al, 2011 (5) | Randomized cross-over study | 16 male collegiate athletes (mean age 21 yrs) undergoing warm-up (20 min) followed by maximal exercise (6 min) at cycle ergometer under conditions of low and high PM (LPM and HPM) exposure | PM freshly generated by diesel engine. Particle counts around 2000/cm^3^ and 300,000/cm^3^ during LPM and HPM exposure, respectively. Similar particle size in LPM and HPM experiments. | Exercise performance (i.e. total work), pulmonary artery pressure | Decreased performance after exercise and increased pulmonary artery pressure after exercise in HPM condition. |
| Gomes et al, 2010  (6) | Randomized repeated- measure study | 10 male endurance-trained runners (mean age 24 years; VO2max: 64.4 ± 4.4 ml •kg^-1^ • min^-1^) undergoing a 8-km time trial run | Control: 20°C - 50% relative humidity (rh)  Control + O3: 20°C - 50% rh + 0.10 ppm O3  Heat: 31°C + 70% rh  Heat +O3: 31°C + 70% rh + 0.10 ppm O3. | Performance, pulmonary function, and subjective  respiratory symptoms | Completion time higher for Heat (32 min 35 s) and Heat ± O3 (33 min 09 s) trials compared to Control ± O3 (30 min 27 s) and Control (30 min 15 s) trials.  No significant changes between pre/post lung function measures or between trials or in symptoms |
| Bräuner et al, 2009 (7) | Randomized, two-factor cross-over study | 29 healthy nonsmoker subjects (20 M, 9 F), mean age 27 years. Experimental conditions: 24-h exposure to particle-rich or FA at rest; 24-h exposure to particle-rich or FA with 90 min cycling at 60–75% maximal intensity after exposure for 15 min and 7.5 h, respectively, then rest | Particle-rich air: 6,169-15,362 particles/cm^3^; PM2.5: 7.0-11.6 microg/m^3^; PM10-2.5: 7.5-15.8 µg/m^3^.  FA: 91-542 particles/cm^3^ | 99mTc-DTPA clearance rate  and plasma and urine concentrations of CC16. Lung function measured repeatedly after exposure and exercise | Moderate exercise increased permeability of the alveolar blood-gas barrier, but PM exposure had no significant additional effect. CC16 and lung function unaffected by PM exposure |
| Graff et al, 2009 (8) | Single-blind, cross-over study | 14 healthy, nonsmoker subjects (6 F, 8 M, mean age 25 years) undergoing a 2-day protocol, with intermittent exercise (15 min rest - 15 min recumbent cycling for 2 h) during exposure to pollutants or filtered air (FA) | Exposure to FA, or coarse PM (averaged concentration 89.0 µg/m^3^, range 23.7–159.6 µg/m^3^) **,** on different days. Some fine PM also present during exposure.  Recumbent exercise workload adjusted to reach ventilation of 20 L • min^-1^ • m^2^BSA^-1^ | Spirometry and DLCO before and after exposure, and 20 h after exposure. Bronchial (BL) and bronchoalveolar lavage (BAL) at 20 h after exposure, to measure: a) cell counts; b) IL-6, IL-8, PGE-2, alpha1-antitrypsin, and total protein levels. Complete and differential cell counts, CRP, catecholamines in blood. | Lung function and DLCO unaffected by coarse PM exposure. Increased PMN in BAL, and slightly decreased monocytes in BL, unchanged mediators, reduced total protein levels after coarse PM exposure.  Unchanged blood cell counts, CRP or catecholamines after exposure. |
| Gong et al, 1988 (9) | Randomized, cross-over study | 15 highly trained cyclists (14 M, 1 F, mean age 23 years) during submaximal heavy exercise followed by maximal sprint after acute placebo or albuterol administration | Exposure to O3 0.21 ppm, or filtered air (FA) during rest and exercise | Spirometry and histamine bronchoprovocation at rest and after exercise, metabolic variables during exercise, symptoms post-exercise. | Compared to FA, O3 reduced FVC, FEV1 and FEF25-75 post-exercise. Submaximal exercise: similar metabolic data and performance under all experimental conditions. Maximal sprint: Lower V’Emax in O3. Albuterol administration did not prevent negative effects of O3.  Chest tightness after exercise in O3. Bronchial reactivity similar under all conditions. |

References

1. Giles LV, Carlsten C, Koehle MS. The pulmonary and autonomic effects of high-intensity and low-intensity exercise in diesel exhaust. Environ Health. 2018 Dec 13;17(1):87. doi: 10.1186/s12940-018-0434-6. PubMed PMID: 30541575; PubMed, Central PMCID: PMC6292001.
2. Giles LV, Brandenburg JP, Carlsten C, Koehle MS. Physiological responses to diesel exhaust exposure are modified by cycling intensity. Med Sci Sports Exerc. 2014;46(10):1999-2006. doi: 10.1249/MSS.0000000000000309.
3. Madden MC, Stevens T, Case M, Schmitt M, Diaz-Sanchez D, Bassett M, Montilla TS, Berntsen J, Devlin RB. Diesel exhaust modulates ozone-induced lung function decrements in healthy human volunteers. Part Fibre Toxicol. 2014;11:37. doi: 10.1186/s12989-014-0037-5.
4. Giles LV, Carlsten C, Koehle MS. The effect of pre-exercise diesel exhaust exposure on cycling performance and cardio-respiratory variables. Inhal Toxicol.2012 Oct;24(12):783-9. doi: 10.3109/08958378.2012.717649. PubMed PMID: 23033992.
5. Cutrufello PT, Rundell KW, Smoliga JM, Stylianides GA. Inhaled whole exhaust and its effect on exercise performance and vascular function. Inhal Toxicol. 2011;23(11):658-667. doi: 10.3109/08958378.2011.604106.
6. Gomes EC, Stone V, Florida-James G. Investigating performance and lung function in a hot, humid and ozone-polluted environment. Eur J Appl Physiol. 2010;110(1):199-205. doi: 10.1007/s00421-010-1485-8.
7. Bräuner EV, Mortensen J, Møller P, Bernard A, Vinzents P, Wåhlin P, Glasius M, Loft S. Effects of ambient air particulate exposure on blood-gas barrier permeability and lung function. Inhal Toxicol. 2009;21(1):38-47. doi: 10.1080/08958370802304735.
8. Graff DW, Cascio WE, Rappold A, Zhou H, Huang YC, Devlin RB. Exposure to concentrated coarse air pollution particles causes mild cardiopulmonary effects in healthy young adults. Environ Health Perspect. 2009 Jul;117(7):1089-94. doi: 10.1289/ehp0900558. Epub 2009 Mar 23. PubMed PMID: 19654918; PubMed Central PMCID: PMC2717135.
9. Gong H Jr, Bedi JF, Horvath SM. Inhaled albuterol does not protect against ozone toxicity in nonasthmatic athletes. Arch Environ Health. 1988 Jan-Feb;43(1):46-53. PubMed PMID: 3355243.
