# Supplementary material for: Respiratory Effects of Exposure to Traffic-Related Air Pollutants During Exercise
Source: Front Public Health. 2020 Dec 11;8:575137. doi: 10.3389/fpubh.2020.575137 (PMC7793908; doi:10.3389/fpubh.2020.575137)
Supplement: Supplementary Table 3 — Field studies in cyclists. [file Table_3.DOCX]

| **Table 3. Field studies-cycling** | | | | | |
| --- | --- | --- | --- | --- | --- |
| **Authors** | **Type of study** | **Subjects** | **Exposure** | **Outcomes** | **Key findings** |
| Laeremans et al, 2018 (1) | Longitudinal observational field study (long-term changes) | 115 non-smoking participants (51 M, 64 F mean age , 36.6 years) with wearable sensors for a week in three different seasons (PASTA Project) | Physical activity (PA) level measured by SenseWear armband.  Exposure to black carbon (BC), a marker of air pollution (AP), measured by microAeth | Long-term effects of PA and BC, and their interaction, on lung function | No significant effects of physical activity and BC on lung function parameters in models excluding the interaction term. When the interaction term was included, significant, negative interaction effects of physical activity and BC exposure on FEV1 (P = 0.07),  the FEV1/FVC ratio (P = 0.03), and FEF25–75 (P = 0.03). Trend for PA-associated improvement in lung function for environmental BC concentration up to 1 µg/m^3^. |
| Laeremans et al, 2018 (2) | Longitudinal  observational  field study (short-term changes) | 122 non-smoking participants (55 M, 67 F, mean age 30 years) with wearable sensors for a week in three different seasons (PASTA Project) | Physical activity (PA) level measured by SenseWear armband.  Exposure to black carbon (BC), a marker of air pollution (AP), measured by microAeth | Short-term effects (2h and 24 h) of PA and BC, and their interaction, on lung function and FeNO | 2-h window: PA caused bronchodilation and improved lung function (FEV1: +15.6 mL; p < 0.05). BC% increase associated with decreased lung function (PEF: -0.10 mL; p < 0.05). The interaction between PA and BC exposure indicated a potential protective effect of PA (p < 0.05). FeNO unchanged. 24-h window: few minor changes. |
| Cole et al, 2018 (3) | Randomized cross-over field study | 38 non-smoking participants (M 28, F 10, mean age 29 years) cycling for 1 h Downtown or in a residential area in Vancouver, BC, Canada | Particulate matter air pollution (PM10, PM2.5, and PM1.) and particle number concentration (PNC) measured by sensor on the bicycle | Lung function, C-reactive protein, interleukin-6, and 8-hydroxy-2′-deoxyguanosine assessed within one hour pre- and  post-trial. | Negative study |
| Cole-Hunter et al, 2018 (4) | Longitudinal observational field study | Non-smoking participants (N= 57, 54% female, mean age 34 years) studied on 4 different mornings at least 5 days apart, in a clinical setting between 2011 and 2014. Diaries for 3 days preceding measurements. Physical activity (PA) estimated based on participants’ diaries | Annual and short-term air pollution exposures, including PM10, particulate matter of 10 to 2.5 μm diameter (PMCoarse), and ozone (O3) at participant residential and occupational addresses. | Lung function (spirometry: FEV1, FVC, SUM) | An increase in 10 µg/m^3^ of annual mean PM10 concentration at the residential address was associated with a 0.3% reduction in FVC (p < 0.01) and a 0.5% reduction in SUM (p < 0.04). Increased annual levels at the residential address of O3 and PM10 were associated with reduced lung function. Little effect of estimated PA compared to mean annual exposure to pollutants. |
| Park et al, 2017 (5) | Cross-over field study | Non-smoking cyclists (24 M8 F, mean age 45.1 years) from the Sacramento area who routinely used cycling for active transportation (≥ 3 d/wk). Two rides on separate days in March-June, 2008, on a high traffic and a low traffic route, respectively. | Ambient air pollution (PM2.5, NOX, NO2, NO, and O3) and meteorological data obtained from National Air Monitoring Stations/ State and Local Air Monitoring Stations (NAMS/SLAMS) in the Sacramento and Davis  Areas. Ultrafine particulate matter (UFPM) was used as a surrogate for exposure to pollutants near a roadway. | Acute changes in lung function after each ride | In the final linear mixed-effect model using median UFPM concentrations as the main exposure, lung function decreased post–ride. Increased levels of UFPM concentrations correlated with decrements in lung function measurements. Considered exposures were short-term. |
| Matt et al. 2016 (6) | Cross-over field study with repeated measures | 29 non-smoking healthy adults (M 15, F 14, mean age: 36 years), undergoing four 2-h exposure scenarios that included either rest or intermittent exercise in high- and low-traffic environments. | Traffic related air pollution (TRAP) assessed by measuring PM2.5 and PM10, black carbon (BC), nitrogen monoxide (NO), nitrogen oxides (NOx) | Measures of respiratory function were collected at three time points during each condition. | Exercise in low-traffic: increase of FEV1, FEV1/FVC and FEF25–75%.  PMcoarse exposure at rest: decrease in FEV1 and FVC.  Exercise during PMcoarse exposure: blunted immediate and delayed negative effects of PM upon pulmonary function. Participants pre-exposed to high TRAP levels showed attenuated responses to exercise. |
| Kubesch et al, 2015 (7) | Cross-over field study with repeated measures | 28 non-smoking healthy adults (M 13, F 15; mean age: 34.4 yrs) undergoing four 2-h exposure scenarios that included either rest or intermittent exercise in high- and low-traffic environments. | Traffic related air pollution (TRAP) assessed by measuring PM2.5 and PM10, black carbon (BC), nitrogen monoxide (NO), nitrogen oxides (NOx) | Spirometry; serum IL-6, blood neutrophil (PMN) and leucocyte (WBC) counts; fractional exhaled NO (FeNO) | Exercise increased spirometric variables and FeNO irrespective of high or low TRAP exposure. Exercise increased WBC, PMN and IL-6. Limited and inconsistent evidence for interaction between exercise and pollutant effects. |
| Cole-Hunter et al, 2015 (8) | Cross-sectional survey | 153 non-smoking adult bicycle commuters (mean age 41 years; 28% female) undergoing questionnaire-based investigation in southeast Queensland (SE QLD; surrounding Brisbane) | Maximum daily mean particle mass concentrations were 37 μg/m^3^ for PM10 and 19 μg/m^3^ for PM2.5. | Perceptions, symptoms, and willingness for specific management strategies of exposure to air pollution | In healthy individuals, the frequency of specific acute respiratory symptoms was higher in-commute and post-commute compared with pre-commute. Higher perception of pollution by females and subjects with predisposition to respiratory disease.  Willingness to adopt preventive strategies if shown to be effective. |
| Cole-Hunter et al, 2013 (9) | Cross-over field study | 35 healthy non-smoking adults (mean age 39 years; 29% female) completed two return trips, one in the condition of their typical route (HIGH) and the other on a pre-determined alternative route of lower proximity to motorised traffic (LOW). | Particle number concentration (PNC) and diameter (PD) monitored in-commute in real-time. Mean PNC was 1.91 x e^4^ ppcc in LOW, and 2.95 × e^4^ ppcc; in HIGH | Data collected immediately pre-commute, and immediately and 3 h post-commute. Acute inflammatory indices of respiratory symptoms (1 to 5), lung function and inflammatory cell analyses in spontaneous sputum. | Compared to HIGH, lower mean frequency of in-commute offensive odour detection (2.1 vs. 2.8; p = 0.019), dust and soot observation (1.7 vs. 2.3; p = 0.038) and nasopharyngeal irritation (1.5 vs. 1.9; p = 0.007) were found after commuting in the LOW route. No change in peak flow rate or inflammatory cells in sputum associated with LOW or HIGH. Higher age and female sex associated with worse symptoms in HIGH. |
| Jarjour et al. 2013 (10) | Cross-over field study | 15 healthy non-smoking adults (age 23–48 yrs, 4 females) recruited to cycle on two routes – low-traffic Bicycle Boulevard and high-traffic route. Pollutant levels monitored by sensors on bycicles. | O3, carbon monoxide (CO), sulfur dioxide (SO2), black carbon (BC), PM10, and PM2.5 | Spirometry before-after exercise | Significantly elevated concentrations of PM2.5 and CO, and borderline significant differences for BC were found on the high-traffic route relative to the low-traffic route. Similar spirometric values pre-post exercise in both settings. |
| Nwokoro et al, 2012 (11) | Observational study | 28 non-smoking commuters in London, 14 cyclists (M 9, F 5, mean age 27 years), 14 non-cyclists (M 5, F 12, mean age 23 years).  Induced sputum (IS)samples collected on a usual working days in both groups | Exposure to black carbon (BC), a marker of air pollution (AP), measured by microAeth 79±9 days after collection of (IS) | IS analyzed for alveolar macrophages (AM) containing BC. Serum cytokine levels measured: IL-1beta, IL-2, IL-6, IL-8, GM-CSF, and TNF-alpha | Exposure to BC during commuting in cyclists 2.6-fold higher than in non-cyclists. BC area in AM: 1.81±0.21 µm^2^ in cyclists; 1.11±0.07 µm^2^ in non-cyclists. Higher serum TNF-alpha concentration in cyclists than non-cyclists (borderline significance), differences in other markers not significant |
| Strak et al. 2010 (12) | Repeated-measure study | 12 healthy non-smoking adults (M 4, F 8, mean age 30 years) cycled a low- and a high-traffic intensity route during morning rush hour in Utrecht, Netherlands for 16 days (total: 80 observations). Mean cycling speed 8 km/h. | Particle number concentration (PNC) was 59% higher in high- than in low-traffic route. Average soot  concentration was 39% higher on the high-traffic route; PM10 concentrations not different. | Spirometry (FVC, FEV1, PEF, FEF25-75). FeNO as a marker of airway inflammation | Lung function at the end of exercise increased non-significantly. Exposure to ultrafine particles and soot during cycling was weakly associated with increased FeNO and decrements in lung function 6 h after exposure |
| Jacobs et al, 2010 (13) | Cross-over study | 38 non-smoking volunteers (mean age 43 years, 26% women) cycled for about 20 min in real traffic and in a laboratory with filtered air | PM2.5, PM10, NO, NO2 and ozone concentrations obtained from fixed measuring stations | FeNO, plasma IL-6, serum Clara cell protein, blood cell counts measured before/after exercise test | Percentage of blood neutrophils increased more after exercise in the road test than after exercise in the clean room. Other variables did not differ significantly between the two scenarios. |
| Bergamaschi et al, 2001 (14) | Observational study | 24 non-smoking volunteers (mean age 28.5 years, 15 women) bicycling for 2 h outdoor | Ozone exposure range 32-103 ppb | Spirometry, serum CC16, NQO1 and GSTM genotypes in WBC DNA, 8-hydroxy-29-deoxyguanosine (8-OHdG, marker of ROS-DNA interaction) | Exercise at O3>80 ppb caused decrease in spirometric variables and increased serum CC16. These changes were significant and 8-OHdG increased only in carriers of both the NQO1*wt* and GSTM-*null* genotypes. |
| Bergamaschi et al, 1999 (15) | Randomized cross-over field study | Non-smoking volunteers (M 12, F 12, age 25-37 years) bicycling in:   1. inner city routes with often jammed traffic; 2. open rural routes. | Volunteers were equipped with a passive-diffusive air sampler, to assess time-weighted average (TWA) concentrations of benzene, toluene, ethylbenzene and xylenes (BTEX) over during 2-h runs | Personal exposure  to aromatic hydrocarbons  Epoxide hydrolase (EH) and  glutathione-S-transferase class m-1 (GSTM1) genotypes (enzymes relevant to the bio-transformation of benzene) | Benzene and toluene in blood, and toluene and xylenes in urine, significantly increased after urban  runs compared to pre-run values. Urinary t,t-muconic acid was significantly higher in post-run samples after both urban and rural runs. A significant relationship was observed between airborne benzene and post-run t,t-muconic acid. Genetically-based metabolic differences also responsible for inter-individual variability of biomarkers. |
| Brunekreef et al (1994) (16) | Longitudinal observational  field study | 29 male cyclists, mean age: 25 yr, exercising heavily during voluntary recreational activities in Netherlands. Measurements obtained 30 min before, and 10-60 min after exercise and competitions over a summer season. | Environmental O3 measured by air monitoring stations, average 87 µg/m^3^, maximum 195 µg/m^3^ | Spirometry (FVC, FEV1, PEF, FEF25-75), respiratory symptoms | Difference between pre-/post-exercise lung function negatively related to the O3 concentration during exercise. Significant decrease observed only for FVC and PEF. The difference between pre- and post-exercise acute symptoms, i.e., shortness of breath, chest tightness, and wheeze, was positively related to O3 concentration. |

References

1. Laeremans M, Dons E, Avila-Palencia I, Carrasco-Turigas G, Orjuela-Mendoza JP, Anaya-Boig E, Cole-Hunter T, DE Nazelle A, Nieuwenhuijsen M, Standaert A, VAN Poppel M, DE Boever P, Int Panis L. Black carbon reduces the beneficial effect of physical activity on lung function. Med Sci Sports Exerc. 2018;50(9):1875-1881. doi: 10.1249/MSS.0000000000001632.
2. Laeremans M, Dons E, Avila-Palencia I, Carrasco-Turigas G, Orjuela JP, Anaya E, Cole-Hunter T, de Nazelle A, Nieuwenhuijsen M, Standaert A, Van Poppel M, De Boever P, Int Panis L. Short-term effects of physical activity, air pollution and their interaction on the cardiovascular and respiratory system. Environ Int. 2018;117:82-90. doi: 10.1016/j.envint.2018.04.040.
3. Cole CA, Carlsten C, Koehle M, Brauer M. Particulate matter exposure and health impacts of urban cyclists: a randomized crossover study. Environ Health. 2018;17(1):78. doi: 10.1186/s12940-018-0424-8.
4. Cole-Hunter T, de Nazelle A, Donaire-Gonzalez D, Kubesch N, Carrasco-Turigas G, Matt F, Foraster M, Martínez T, Ambros A, Cirach M, Martinez D, Belmonte J, Nieuwenhuijsen M. Estimated effects of air pollution and space-time-activity on cardiopulmonary outcomes in healthy adults: A repeated measures study. Environ Int. 2018;111:247-259. doi: 10.1016/j.envint.2017.11.024.
5. Park HY, Gilbreath S, Barakatt E. Respiratory outcomes of ultrafine particulate matter (UFPM) as a surrogate measure of near-roadway exposures among bicyclists. Environ Health. 2017;16(1):6. doi: 10.1186/s12940-017-0212-x.
6. Matt F, Cole-Hunter T, Donaire-Gonzalez D, Kubesch N, Martínez D, Carrasco-Turigas G, Nieuwenhuijsen M. Acute respiratory response to traffic-related air pollution during physical activity performance. Environ Int. 2016;97:45-55. doi: 10.1016/j.envint.2016.10.011
7. Kubesch NJ, de Nazelle A, Westerdahl D, Martinez D, Carrasco-Turigas G, Bouso L, Guerra S, Nieuwenhuijsen MJ. Respiratory and inflammatory responses to short-term exposure to traffic-related air pollution with and without moderate physical activity. Occup Environ Med. 2015 Apr;72(4):284-93. doi: 10.1136/oemed-2014-102106. Epub 2014 Dec 4. PubMed PMID: 25475111.
8. Cole-Hunter T, Morawska L, Solomon C. Bicycle commuting and exposure to air pollution: a questionnaire-based investigation of perceptions, symptoms, and risk management strategies. J Phys Act Health. 2015 Apr;12(4):490-9. doi: 10.1123/jpah.2013-0122.
9. Cole-Hunter T, Jayaratne R, Stewart I, Hadaway M, Morawska L, Solomon C. Utility of an alternative bicycle commute route of lower proximity to motorized traffic in decreasing exposure to ultra-fine particles, respiratory symptoms and airway inflammation--a structured exposure experiment. Environ Health. 2013;12(1):29. doi: 10.1186/1476-069X-12-29.
10. Jarjour S, Jerrett M, Westerdahl D, de Nazelle A, Hanning C, Daly L, Lipsitt J, Balmes J. Cyclist route choice, traffic-related air pollution, and lung function: a scripted exposure study. Environ Health. 2013;12:14. doi: 10.1186/1476-069X-12-14.
11. Nwokoro C, Ewin C, Harrison C, Ibrahim M, Dundas I, Dickson I, Mushtaq N, Grigg J. Cycling to work in London and inhaled dose of black carbon. Eur Respir J. 2012 Nov;40(5):1091-7. doi: 10.1183/09031936.00195711. Epub 2012 Feb 23. PubMed PMID: 22362851.
12. Strak M, Boogaard H, Meliefste K, Oldenwening M, Zuurbier M, Brunekreef B, Hoek G. Respiratory health effects of ultrafine and fine particle exposure in cyclists. Occup Environ Med. 2010;67(2):118-124. doi: 10.1136/oem.2009.046847.
13. Jacobs L, Nawrot TS, de Geus B, Meeusen R, Degraeuwe B, Bernard A, Sughis M, Nemery B, Panis LI. Subclinical responses in healthy cyclists briefly exposed to traffic-related air pollution: an intervention study. Environ Health. 2010 Oct 25;9:64. doi: 10.1186/1476-069X-9-64. PubMed PMID: 20973949; PubMed Central PMCID: PMC2984475.
14. Bergamaschi E, De Palma G, Mozzoni P, et al. Polymorphism of quinone-metabolizing enzymes and susceptibility to ozone-induced acute effects. Am J Respir Crit Care Med. 2001;163(6):1426–1431. doi:10.1164/ajrccm.163.6.2006056
15. Bergamaschi E, Brustolin A, De Palma G, Manini P, Mozzoni P, Andreoli R, Cavazzini S, Mutti A. Biomarkers of dose and susceptibility in cyclists exposed to monoaromatic hydrocarbons. Toxicol Lett. 1999 Sep 5;108(2-3):241-7. PubMed PMID: 10511268.
16. Brunekreef B, Hoek G, Breugelmans O, Leentvaar M. Respiratory effects of low-level photochemical air pollution in amateur cyclists. Am J Respir Crit Care Med. 1994;150(4):962-966.
