# Supplementary material for: Respiratory Effects of Exposure to Traffic-Related Air Pollutants During Exercise
Source: Front Public Health. 2020 Dec 11;8:575137. doi: 10.3389/fpubh.2020.575137 (PMC7793908; doi:10.3389/fpubh.2020.575137)
Supplement: Supplementary Table 4 — Field studies in runners, soccer players and hikers. [file Table_4.DOCX]

| **Table 4. Field studies runners-soccer players-hikers** | | | | | |
| --- | --- | --- | --- | --- | --- |
| **Authors** | **Type of study** | **Subjects (number)** | **Exposure** | **Outcomes** | **Key findings** |
| Pun & Ho, 2019 (1) | Randomized cross-over study-acute exercise | Healthy nonsmoking habitual runners (13 M, 17 F, mean age 20.6 years)  30-min run at 2 sites | Site 1: ozone 73 ± 66 ppb, black carbon (BC) 5.4 ± 1.6 µg/m^3^  Site 2: ozone 58 ± 60 ppb, BC 1.3 ± 1.7 µg/m^3^ | spirometry | No effects of short-term exposure to pollutants during exercise on respiratory variables |
| Boussetta et al, 2017 (2) | Randomized cross-over study-acute exercise | 11 healthy nonsmoking soccer players (mean age 21.8 years) performing Yo-Yo Intermittent Recovery Test Level-1 (YYIRT1) at two different times of day (08:00 h and 18:00 h) | YYIRT1 in two areas, polluted (PA, Air quality index >200) and non-polluted (NPA, Air quality index <50). Multiple environmental pollutants | Respiratory and metabolic responses to exercise | VO2max, red blood cells (RBC), hemoglobin (Hb), pH, and bicarbonate levels decreased post-YYIRT1 in PA compared to NPA. White blood cell neutrophil, and lymphocyte counts, and PaCO2 levels were higher in PA compared to NPA. |
| Cavalcante de Sá et al, 2016 (3) | Randomized cross-over study during training | 38 young nonsmoking male athletes (age range 18-22 years) ran for 45 min d^-1^ randomly during 5 consecutive days, with an interval of 48 h between Street and Forest environment. Evaluation before and after the first run on Mondays (day 1) and Fridays (day 5) | Street: mean PM2.5 concentration 65.1 ± 39.1 μg m^-3^, temperature T 22.0°C  Forest: mean PM2.5 concentration 22.6 ± 15.3 μg m^-3^, T 22.8 °C | Nasal mucociliary clearance (MCC); pH of exhaled breath condensate (EBC); cell counts and differentials in nasal fluid lavage (NFL); IL-8 and IL-10 in EBC and NFL | At day 5, the number of athletes with impaired nasal MCC was two-fold in the Street group compared to the Forest group. EBC pH increased in the Forest group, with significant differences between groups at day 1 and 5. The number of cells in the nasal lavage fluid NFL was reduced after exercise, without alterations in cell type or IL-8/ IL-10 concentrations irrespective of Street or Forest group. |
| Bos et al, 2013 (4) | Parallel-group study during training | Two groups of untrained nonsmoking subjects undergoing aerobic training for 12 wk, 3 sessions a week: one group in an urban environment M 4, F 11, age 28±8 yr) and another group (M 5, F 4, age 39±6 yr) in a rural environment. | Ultrafine PM (UFPM) concentrations were measured during each training session. Mean UFPM: 7244±2559/cm^3^ and 5625± 1896/cm^3^  at urban and rural location, respectively | Effect of UFPM exposure during aerobic training on: markers of systemic (differential leukocyte counts) and respiratory inflammation (exhaled nitric oxide, eNO). | Fitness levels improved equally in both groups. Leukocyte counts, neutrophil counts, and eNO increased after training in the urban group, while did not change in the rural group. Positive correlations were found between changes in biomarkers and individual exposure level to UFMP |
| Marr & Ely, 2010 (5) | Analysis of marathon race performances | Top performances of the first three men and women from seven competitive US marathon races over several years | Pollutant levels measured during the day of the race: CO, O3, PM10,  PM2.5, NO2, SO2 | To quantify decrements in performance associated with air pollutants, and examine potential sex differences | Levels of pollutants during 168 race-years of US marathons rarely exceeded the EPA health limits, or the levels known to affect lung function. PM10 was associated with reduced performance in women. For every 10-µg/m^3^ increase in PM10, performance decreased by 1.4%. |
| Blair et al, 2010 (6) | Observational study-acute exercise | 10 non-elite nonsmoking athletes (9 M, 1 F, mean age 21.1 years) ran for 20 min along 100 m near a busy roadway. Exercise intensity: 93±2.3% HRmax. | Environmental levels: toluene (53.1 ± 4.2 µg/m^3^), ethylbenzene (428 ± 83 µg/m^3^) and xylene (80.0 ±3.7 μg/m^3^) | Blood levels of benzene, toluene, ethylbenzene and xylene (BTEX) determined pre- and post-exercise. Ventilation during exercise measured in the laboratory during exercise of similar intensity and duration. | Significant increases post-exercise in blood levels for toluene (mean increase of 1.4 ng/ml; p=0.002), ethylbenzene (0.7 ng/ml; p=0.0003), m/p-xylene (2.0 ng/ml; p=0.004) and o-xylene (1.1 ng/ml; p=0.002).  Ventilation during exercise: 86.2 ±2.3 L/min. |
| Chimenti et al, 2009 (7) | Observational repeated-measure study-acute exercise | 9 male non-smoking amateur runners (age 40±4 yr) regularly training outdoors in an urban environment studied at rest (baseline) and the morning after races held in the Fall (21 km), Winter (12 km), and Summer (10 km). | Temperature, humidity or airborne pollutants (O3 and PM10) were monitored during the week preceding the race and during the day of the race. Temperature, NO2 and O3 were higher during Summer than in Fall and Winter. Air pollutant concentrations below the alert threshold at all times. | Cell composition, apoptosis, and inflammatory mediators (TNF-α, IL-8) were measured in induced sputum (IS) | In IS, apoptosis of neutrophils (PMN) increased with O3 and PM10 exposure. Bronchial epithelial cell (BEC) counts were low at all times and weakly correlated with O3 and PM10 levels. PMN counts not related with TNF-α or IL-8 at 20 h after race. Apoptosis of PMNs increased with exposure to environmental pollutants, while apoptosis of BECs increased after intense exercise. |
| Rundell et al, 2008 (8) | Randomized cross-over study-acute exercise | 12 physically fit non-asthmatic nonsmoking males (mean age 20.5 years) studied at rest and after 30-min running at 85-90% of maximal heart rate (2 outdoor trials, 4-5 days apart, in high- and low-pollution areas) | Low-pollution (PM1: 7,382 ± 1,727 particles/cm^3^)  High-pollution (PM1: 252,290 ± 77,529 particles/cm^3^) | Measurements pre-post exercise: spirometry; EBC collection for S -Nitrosoglutathione, nitrate, and (MDA) determination; exhaled NO for NO flux and fractional alveolar contribution | After exercise in the high-pollution area, FEV1 and FEF25-75 decreased; EBC NO3 decreased, and MDA increased; eNO was unaffected, but the alveolar NO contribution decreased. Such changes did not occur after exercise in low-pollution area. Significant relationships were found between spirometric changes after exercise and PM1 concentrations. |
| Ferdinands et al, 2008 (9) | Observational repeated-measure study during training | 16 adolescent runners (mean age 14.9 years, 56% male) daily running long-distance for ten days during peak smog season | Ozone concentration  -Mean ambient 1-hour maximum: 71 ±18 ppb  -Median (interquartile range, IQR):61 (54–67) ppb.  PM2.5 concentration  Mean at 5 p.m.: 27 ± 12 μg/m^3^  Median (IQR): 23.2 (21.7– 34.7) μg/m^3^ | Breath pH | No association between ozone or PM2.5 and post-exercise breath pH. Breath pH low in runners compared to a control sample of 14 relatively sedentary healthy adults |
| Korrick et al, 1998 (10) | Cross-sectional study-acute exercise | 533 volunteer nonsmoking hikers (18-64 years of age) at Mt. Washington, NH during two summer periods (78 days in 1991-1992) | Exposure recorded at base and summit.  Ozone (O3) hourly concentration: mean 40±12 ppb, range 21-74 ppb.  Fine particulate matter (PM2.5): mean daily concentration 10 µg/m^3^, maximum 60 µg/m^3^.  Strong aerosol acidity: median 0.3 µg/m^3^, maximum 20 µg/m^3^. | Pulmonary function measured before and after hiking (on average 8.0±1.5 h) | For each 50 ppb increment in mean O3 FEV1 declined by 2.6%, and FVC declined by 2.2%. There were consistent decrements in both FVC and PEFR with PM2.5 and decrements in PEFR with strong aerosol acidity. Hikers with a history of asthma or wheeze had significantly greater air pollution-related changes in pulmonary function compared to non-asthmatic subjects. |
| Kinney et al, 1996 (11) | Observational repeated-measure study -acute exercise | 19 nonsmoking volunteer joggers (18 M, 1 F; age range 23-38 years) who exercised in the afternoon during the 1992 Summer season in Governors Island, NY. Fifteen subjects re-tested during the following, low ozone, Winter season, and 6 in the following Summer | Average Summer-Winter-Summer O3 values: 58-32-69 ppb. Stable values of NO2 (32-33-33 ppb), and PM10 (27-24-28 µg/m^3^) | Bronchoalveolar lavage (BAL): cell differentials, release of IL-8, TNF-α, and reactive oxygen species (ROS) by pooled cells, and levels of IL-8, protein, LDH, fibronectin,  a1-antitrypsin (a1-AT), complement fragment 3a (C3a), and prostaglandin E2 (PGE2) in lavage fluids. | Release of ROS by stimulated BAL cells lower in Summer than in Winter (p =0.03). LDH levels in BAL were 2-fold higher in Summer than in Winter (p = 0.02), as were IL-8 (p = 0.12) and PGE2 (p = 0.06). |

References

1. Pun VC, Ho KF. Blood pressure and pulmonary health effects of ozone and black carbon exposure in young adult runners. Sci Total Environ. 2019 Mar 20;657:1-6. doi: 10.1016/j.scitotenv.2018.11.465. Epub 2018 Nov 30. PubMed PMID: 30530214.
2. Boussetta N, Abedelmalek S, Aloui K, Souissi N. The effect of air pollution on diurnal variation of performance in anaerobic tests, cardiovascular and hematological parameters, and blood gases on soccer players following the Yo-Yo Intermittent Recovery Test Level-1. Chronobiol Int. 2017;34(7):903-920. doi: 10.1080/07420528.2017.1325896.
3. Cavalcante de Sá M, Nakagawa NK, Saldiva de André CD, Carvalho-Oliveira R, de Santana Carvalho T, Nicola ML, de André PA, Nascimento Saldiva PH, Vaisberg M. Aerobic exercise in polluted urban environments: effects on airway defense mechanisms in young healthy amateur runners. J Breath Res. 2016 Dec 21;10(4):046018. doi: 10.1088/1752-7163/10/4/046018. PubMed PMID: 28000620.
4. Bos I, De Boever P, Vanparijs J, Pattyn N, Panis LI, Meeusen R. Subclinical effects of aerobic training in urban environment. Med Sci Sports Exerc. 2013 Mar;45(3):439-47. doi: 10.1249/MSS.0b013e31827767fc. PubMed PMID: 23073213.
5. Marr LC, Ely MR. Effect of air pollution on marathon running performance. Med Sci Sports Exerc. 2010 Mar;42(3):585-91. doi: 0.1249/MSS.0b013e3181b84a85. PubMed PMID: 19952812.
6. Blair C, Walls J, Davies NW, Jacobson GA. Volatile organic compounds in runners near a roadway: increased blood levels after short-duration exercise. Br J Sports Med 2010; 44:731–735. doi:10.1136/bjsm.2008.051888
7. Chimenti L, Morici G, Paterno A, Bonanno A, Vultaggio M, Bellia V, Bonsignore MR. Environmental conditions, air pollutants, and airway cells in runners: a longitudinal field study. J Sports Sci. 2009 Jul;27(9):925-35. doi: 10.1080/02640410902946493. PubMed PMID: 19629842.
8. Rundell KW, Slee JB, Caviston R, Hollenbach AM. Decreased lung function after inhalation of ultrafine and fine particulate matter during exercise is related to decreased total nitrate in exhaled breath condensate. Inhalation Toxicology 2008; 20: 1-9, DOI: 10.1080/08958370701758593
9. Ferdinands JM, Crawford CA, Greenwald R, Van Sickle D, Hunter E, Teague WG. Breath acidification in adolescent runners exposed to atmospheric pollution: a prospective, repeated measures observational study. Environ Health. 2008 Mar 7;7:10. doi: 10.1186/1476-069X-7-10. PubMed PMID: 18328105; PubMed Central PMCID: PMC2292713.
10. Korrick SA, Neas LM, Dockery DW, Gold DR, Allen GA, Hill LB, Kimball KD, Rosner BA, Speizer FE. Effects of ozone and other pollutants on the pulmonary function of adult hikers. Environ Health Perspect. 1998 Feb;106(2):93-9. PubMed PMID: 9435151; PubMed Central PMCID: PMC1533017.
11. Kinney PL, Nilsen DM, Lippmann M, Brescia M, Gordon T, McGovern T, El-Fawal H, Devlin RB, Rom WN. Biomarkers of lung inflammation in recreational joggers exposed to ozone. Am J Respir Crit Care Med. 1996 Nov;154(5):1430-5. PubMed PMID: 8912760.
